# NKG7 is a Stable Marker of Cytotoxicity Across Immune Contexts and Within the Tumor Microenvironment

**DOI:** 10.1002/eji.202551885

**Published:** 2025-06-20

**Authors:** Roberta Turiello, Susanna S. Ng, Elisabeth Tan, Gemma van der Voort, Nazhifah Salim, Michelle C. R. Yong, Malika Khassenova, Johannes Oldenburg, Heiko Rühl, Jan Hasenauer, Laura Surace, Marieta Toma, Tobias Bald, Michael Hölzel, Dillon Corvino

**Affiliations:** ^1^ Institute of Experimental Oncology Medical Faculty University Hospital Bonn University of Bonn Bonn Germany; ^2^ Institute of Clinical Chemistry and Clinical Pharmacology University Hospital Bonn University of Bonn Bonn Germany; ^3^ Institute for Experimental Hematology and Transfusion Medicine University Hospital Bonn University of Bonn Bonn Germany; ^4^ Computational Health Center Helmholtz Zentrum München Deutsches Forschungszentrum für Gesundheit und Umwelt (GmbH) Neuherberg Germany; ^5^ Faculty of Mathematics and Natural Sciences and the Life and Medical Sciences Institute (LIMES) Rheinische Friedrich‐Wilhelms‐Universität Bonn Bonn Germany; ^6^ Institute of Pathology University Hospital Bonn. University of Bonn Bonn Germany

**Keywords:** cytotoxicity, CD8+ T‐cells, granzyme B, NKG7, natural killer cells, single‐cell RNA sequencing, tumor microenvironment

## Abstract

Cytotoxicity is a cornerstone of immune defense, critical for combating tumors and infections. This process relies on the coordinated action of granzymes and pore‐forming proteins, with granzyme B (GZMB) and perforin (*PRF1*) being key markers and the most widely studied molecules pertaining to cytotoxicity. However, other human granzymes and cytotoxic components remain underexplored, despite growing evidence of their distinct, context‐dependent roles. Natural killer cell granule protein 7 (NKG7) has recently emerged as a crucial cytotoxicity regulator, yet its expression patterns and function are poorly understood. Using large publicly available single‐cell RNA sequencing atlases, we performed a comprehensive profiling of cytotoxicity across immune subsets and tissues. Our analysis highlights NKG7 expression as a strong marker of cytotoxicity, exhibiting a strong correlation with overall cytotoxic activity (*r* = 0.97) and surpassing traditional markers such as granzyme B and perforin in reliability. Furthermore, NKG7 expression is notably consistent across diverse immune subsets and tissues, reinforcing its versatility and robustness as a cytotoxicity marker. These findings position NKG7 as an invaluable tool for evaluating immune responses and a reliable indicator of cytotoxic functionality across biological and clinical contexts.

## Graphical abstract

Cytotoxicity‐associated genes exhibit heterogeneity at the cellular and tissue levels (left). NKG7 gene expression is strongly associated with a cytotoxic gene signature (middle). NKG7 expression is stable and consistently detected in cells across immunologically relevant tissues and within tumor‐infiltrating immune cells (right). Figure generated in collaboration with Susan Stone (https://www.sue‐stone.com).

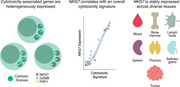

## 1 Introduction

Immunotherapy has revolutionized cancer treatment, significantly improving patient prognosis and, in some cases, offering curative potential. Most immunotherapy strategies leverage the antitumor capabilities of CD8+ T‐cells and Natural Killer (NK) cells. These cells exert antitumor functions through the release of specific cytokines, death‐receptor signaling, or the release of cytotoxic granules. The deployment of cytotoxic granules is especially critical, as they induce targeted, rapid apoptosis of target cells. Cytotoxic granules are specialized secretory lysosomes that release their cytotoxic payload into the immunological synapse, consisting of granzymes, perforin, and granulysin [1].

Granzymes are a core component of the cytotoxic granule‐mediated death machinery. These serine proteases cleave various intracellular substrates to initiate target‐cell death. In humans, five granzymes (A, B, H, K, and M) have been identified, each with differing substrate specificities and thus, the capacity to induce distinct forms of cell death [2]. However, the contexts, heterogeneity, and dynamics of granzyme expression remain poorly understood. Originally considered redundant, granzymes are now increasingly recognized for their distinct and specialized functions. For example, granzyme A and B—the most well‐studied members of the granzyme family—induce caspase‐independent pyroptosis and caspase‐dependent apoptosis respectively [3, 4, 5, 6]. Meanwhile, granzymes H, K, and M remain poorly understood but exhibit unique functions, including the induction of alternative apoptosis pathways, microtubule disruption, cytokine processing, extracellular matrix remodeling, and modulating inflammatory responses [7, 8]. These latter processes highlight some of the noncytotoxic functionalities increasingly being attributed to granzyme activity. Perforin is a key mediator of cytotoxicity and predominantly functions to facilitate the delivery of cytotoxic effectors such as granzymes and granulysin into target cells [9]. Granulysin is involved in cytotoxicity and antimicrobial response, contributing to antitumoral responses through membrane disruption and immune modulation [10]. Together, these granule components orchestrate the rapid and efficient cytotoxic response while engaging in noncytotoxic roles that can be either pro‐tumorigenic or anti‐inflammatory.

Tumors and pathogens have evolved mechanisms to evade cytotoxicity, including overexpressing specific granzyme inhibitors or downregulating granzyme targets, thus reducing their susceptibility to particular granzymes [11]. This highlights the potential benefits of granzyme heterogeneity. For instance, granzyme B is selectively inhibited by molecules such as Serpin‐B9, while the other granzymes are unaffected [12]. Similarly, granzyme H degrades an adenoviral inhibitor of granzyme B [13]. This evolutionary interplay underscores the necessity for the diverse yet overlapping functions of the granzyme family.

In recent years, natural killer cell granule protein 7 (NKG7) has emerged as a potent marker of cytotoxic populations. Increasingly, *NKG7* expression is used to identify cytotoxic populations in sequencing datasets; however, research into the expression and immunological role of NKG7 is in its infancy. Specifically, the function and expression dynamics of NKG7 are largely unknown. NKG7 was first identified to be expressed in the cytotoxic granules of NK and T cells and has subsequently been shown to regulate antitumoral effector functions [14, 15, 16, 17, 18]. It is thought that NKG7 is involved in the release of cytotoxic granules during the immune response [14, 16, 19]. Clinically, NKG7 expression is associated with improved patient outcomes across various tumor entities [14, 20, 21, 22, 23]. Altogether, current findings demonstrate the importance of NKG7 in regulating antitumoral cytotoxicity and its utility for the assessment and prediction of clinical responses to immunotherapy.

Granzyme functions are diverse, yet much remains unknown about this heterogeneity and it is often underappreciated. The majority of research has focused on granzymes A and B, overlooking the broader diversity of granzyme functions. NKG7, a newly emerging player in cytotoxicity, shows promise as a strong correlate of cytotoxic activity. In our study, we comprehensively profiled cytotoxic molecule usage across cytotoxic and noncytotoxic populations in healthy patients, various tissues, and disease settings. We found NKG7 expression outperforms traditional markers of cytotoxicity, such as granzyme B and perforin, in capturing cytotoxic populations. Furthermore, NKG7 was found to be stably expressed across tissues, cell subsets, and disease conditions. While we confirmed some expected expression patterns such as the expression of granzyme B in pDCs, we also identified previously overlooked markers [24]. For example, a notable proportion of effector cells lack significant perforin expression. Our findings suggest NKG7 may serve as a valuable pan‐cytotoxicity marker, crucial for identifying cytotoxic cells despite inherent granzyme heterogeneity.

## 2 Results

### 2.1 Cytotoxicity Profiling Reveals Conserved and Distinct Patterns of Cytotoxic Molecule Expression in Human PBMCs

**FIGURE 1:**
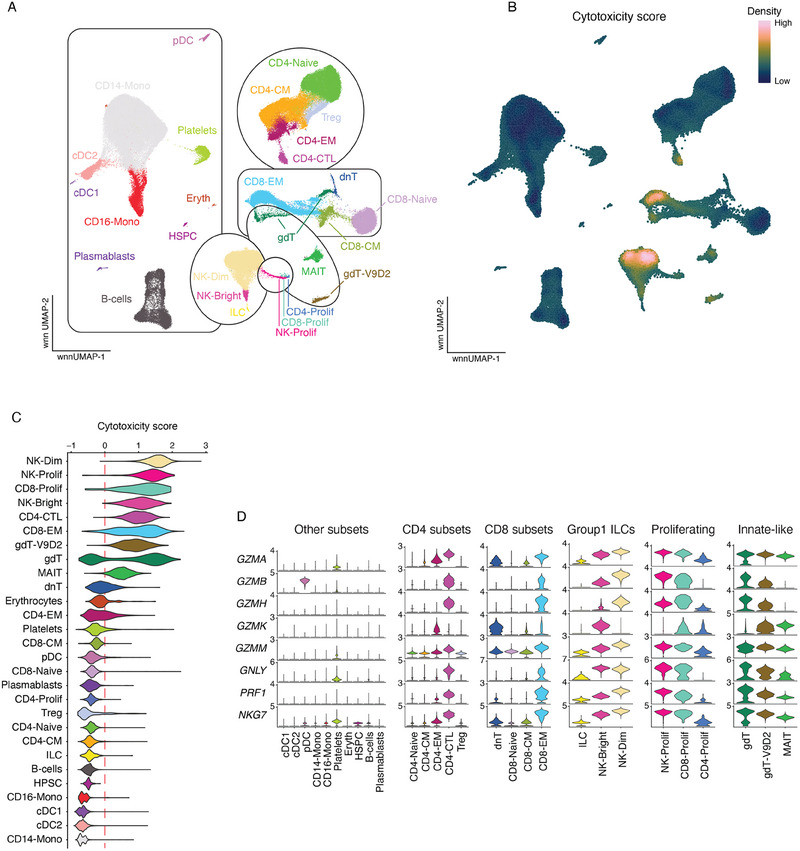
Pattern of cytotoxic molecule expression in human PBMC subsets. (A) The cellular subsets of human PBMC as identified using integrated scRNAseq and scCITEseq data visualized on weighted‐nearest neighbor (wnn) UMAP coordinates. (B) The density of expression for cytotoxicity score overlayed on wnnUMAP. (C) Violin plots showing the “cytotoxicity score” across immune populations. (D) Violin plots showing the imputed expression of cytotoxicity markers that contribute to the cytotoxicity score. Cellular subsets are abbreviated as follows: CD14⁺ monocyte (CD14‐Mono), CD16⁺ monocyte (CD16‐Mono), CD56BrightCD16⁻ (NK‐Bright), CD56DimCD16⁺ (NK‐Dim), central memory (CM), conventional dendritic cell type 1 (cDC1), conventional dendritic cell type 2 (cDC2), cytotoxic T‐lymphocyte (CTL), double‐negative T‐cell (dnT; CD4⁻CD8⁻ T‐cells), effector memory (EM), gamma delta T‐cell (gdT), hematopoietic stem and progenitor cell (HPSC), innate lymphoid cell (ILC), mucosal‐associated invariant T‐cell (MAIT), plasmacytoid dendritic cell (pDC), proliferating (Prolif), regulatory T‐cell (Treg), Vγ9Vδ2 gamma delta T‐cells (gdT‐V9D2).

To investigate cytotoxic molecule expression, we utilized a scRNAseq dataset of peripheral blood mononuclear cells (PBMCs) from healthy donors [25]. This dataset, derived from multimodal RNA and protein sequencing, encompasses well‐defined cell subsets (Figure 1A). Scoring cells for cytotoxicity‐associated transcripts (*GZMA*, *GZMB*, *GZMH*, *GZMK*, *GZMM*, *GNLY*, *PRF1*, and *NKG7*) reveals a high density of cytotoxic molecule expression in NK‐Dim (CD56DimCD16+) and CD8‐EM (Effector Memory) (Figure 1B–C). Inspection of the cytotoxicity score reveals NK‐Dim, proliferative NK, and CD8 populations as high expressers of cytotoxicity markers (Figure 1C). Despite being classically described as an immature NK cell subset and pro‐inflammatory subset [26], NK‐Bright (CD56BrightCD16‐) cells scored highly for cytotoxicity, with levels similar to what is seen in classical cytotoxic populations such as CD4‐CTLs and CD8‐EM cells. Innate‐like populations also showed diverse levels of cytotoxicity‐associated transcripts ranging from highly cytotoxic (gdT‐V9D2) to poorly cytotoxic (MAIT) populations. Strikingly, gdT cells exhibited a bimodal cytotoxicity distribution with sub‐populations both high and low in cytotoxicity (Hartigan's dip test, *p* < 2.2e‐16). To further probe these results, the expression of individual markers was evaluated across immune subsets (Figure 1D). This interrogation revealed both expected and poorly described or seldom appreciated granzyme expression patterns. For example, the classic cytotoxic subsets (CD8‐EM, NK‐Dim, and CD4‐CTLs) all had similar and expected expression patterns. These subsets expressed high levels of all cytotoxic molecules except *GZM*K, which was low or absent. A similar expression pattern was observed in gdT and proliferating NK subsets. Conversely, NK‐bright cells, which are typically described as pro‐inflammatory or regulatory in nature, expressed high levels of all markers except *GZMH*. Interestingly, the gdT‐V9D2 population could be distinguished from the remaining gdT cells by the expression of *GZMK*. Additionally, gdT‐V9D2 along with CD8‐Prolif cells were the only two populations assessed that showed appreciable levels of expression of all cytotoxic molecules. Meanwhile, MAIT cells expressed high levels of all cytotoxic molecules except *GZMB* and *GZMH*. This reveals that the low cytotoxicity score observed for MAIT cells is driven by the lack of *GZMB* and *GZMH*. Of note, pDCs displayed high expression of *GZMB* but no other cytotoxic molecule. Additionally, a low level of expression of *GZMA*, *GZMM*, *GNLY*, and *NKG7* was detected in platelets. Evaluation of the cytotoxic landscape of human PBMCs revealed known and unexpected expression patterns for cytotoxic molecules. This analysis reveals that populations not typically considered cytotoxic can express appreciable levels of cytotoxic molecules, while certain immunological subsets appear to prefer the expression of particular cytotoxic molecule patterns. However, the transcript level may not reflect the functional cytotoxic activity in vivo, and further investigations are needed.

### 2.2 NKG7 is a Reliable Marker of Cytotoxicity in Human PBMC Subsets

**FIGURE 2:**
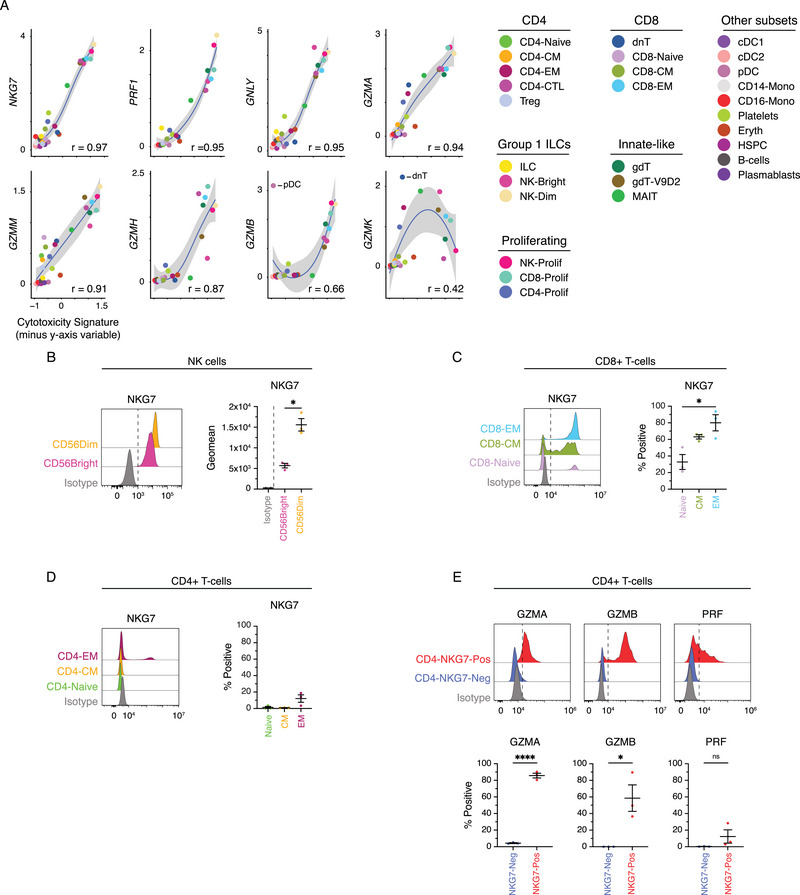
NKG7 correlates with cytotoxicity across human PBMC subsets. (A) Scatter plots demonstrating the correlation between *y*‐axis gene expression and cytotoxicity signature across PBMC subsets. Pearson correlation is displayed and the shaded area represents the 95% CI. (B) Histograms showing the expression of NKG7 in NK cell populations and the corresponding geometric mean fluorescence intensity in CD56Bright and CD56Dim populations. *p*‐values from Welch's *t‐*test. (C–D) Histograms showing the expression of NKG7 in EM, CM, and Naïve CD8+ T‐cells or EM, CM, and Naïve CD4+ T‐cells, respectively; and dot plots showing the frequency of NKG7+ populations. *P* values from ordinary one‐way ANOVA. (E) Histograms showing the expression of GZMA, GZMB, PRF1, and in NKG7 positive and negative CD4+ T‐cells, and corresponding dot plots indicating the frequency of positive cells. Dashed lines in the histograms do not represent gating thresholds but are included for visual comparison. Gating was determined based on unstained controls, isotype controls, or fluorescence minus one (FMO) controls, depending on the most appropriate approach for each marker. *p*‐values from unpaired *t*‐test. *p < 0.05; ****p value < 0.0001.

Given the diverse patterns of cytotoxic molecule expression observed in human PBMCs, we sought to further evaluate the dynamics of cytotoxic molecule use. The correlation of each cytotoxic gene with one another was evaluated within the two most cytotoxic subsets (CD8 and NK cells). This revealed *GZMK* as poorly and inversely correlated with the expression of other cytotoxic genes in both CD8 and NK subsets (Figure S1A). This prompted us to ask which cytotoxic gene is most strongly and consistently correlated with cytotoxicity across all subsets present within human PBMCs. To evaluate this, subsets were iteratively scored for cytotoxicity using a signature of all cytotoxic genes except the gene of interest. The correlation between the gene of interest and the overall cytotoxicity score was then determined and visualized. The analysis revealed that *NKG7* expression had the strongest correlation (*r* = 0.97) with cytotoxicity score (Figure 2A). *PRF1*, *GNLY*, and *GZMA* (*r* = 0.95, *r* = 0.95, *r* = 0.94; respectively) all had similar correlation values with cytotoxicity score. Interestingly, *GZMB* had the second lowest correlation score (*r* = 0.66), which was driven by the unique and singular expression of *GZMB* in pDCs. Excluding pDC cells from analysis resulted in a correlation score of *r* = 0.9 for *GZMB* (Figure S1B).

Given these findings and the known discordance of RNA and protein, we sought to investigate the expression patterns of NKG7 at the protein level. Interestingly, limited studies have evaluated the protein expression of NKG7, perhaps due to limited reagent availability. As such, we utilized the anti‐TIA1 antibody clone 2G9A10F5 (herein referred to as 2G9) [15]. This monoclonal antibody recognizes a pentameric epitope (GYETQ) at the C‐terminus of TIA1. Similarly, the C‐terminus of NKG7 contains the pentameric GYETL sequence (Figure S1C). Others have established 2G9 as a cross‐reactive antibody capable of binding both NKG7 and TIA1 [15]. We validated these findings using transfected HEK293T cells over‐expressing tagged TIA‐1 or NKG7. Western blot analysis confirmed that 2G9 is cross‐reactive for NKG7 and TIA‐1 (Figure S1D). *NKG7* and *TIA1* genes are encoded on separate chromosomes, with *NKG7* on chromosome 19 and *TIA1* on chromosome 2. Furthermore, these genes have different expression patterns, predicted structures, and functions. Therefore, *TIA1* is not expected at appreciable levels within immunological subsets. To verify this, we evaluated the expression of *NKG7* or *TIA1* in total or sorted PBMC subsets using both bulk and single‐cell RNAseq datasets (Figure S1E–G). This revealed that minimal to no *TIA1* transcript can be detected across immunological subsets. This observation was further validated at the protein level where TIA1 was not detected in PBMC lysates incubated with the anti‐TIA‐1 antibody EPR9304 (Figure S1H). Although the expression of TIA1 in PBMCs cannot be completely excluded, these results suggest that the signal from 2G9 in PBMCs derives mainly from NKG7.

Hence, we evaluated the protein expression of NKG7 in cytotoxic subsets from healthy PBMC samples using the 2G9 antibody. This verified NK cells as potent expressers of NKG7 with both CD56Bright and CD56Dim populations expressing NKG7. However, on a per‐cell basis, CD56Dim NK cells exhibited significantly higher NKG7 expression levels compared with CD56Bright cells (Figure 2B). In line with transcriptomic data, the frequency of NKG7 expression in CD8 T‐cells increased with differentiation state (Figure 2C). Similarly, NKG7 protein expression in CD4 T‐cells mirrored transcriptomic trends, with the increased levels observed in the CD4‐EM subset (Figure 2D). However, this increase did not reach statistical significance, as only a small proportion (∼12%) of CD4‐EM cells were positive for NKG7. Interestingly, phenotypic identification of CD4‐CTLs is still a matter of debate [27, 28]. However, *NKG7* has appeared in numerous transcriptomic signatures of CD4‐CTLs. [29]. Indeed, we found that NKG7‐positive CD4 T‐cells were enriched for cytotoxic molecules such as GZMA, GZMB, and PRF (Figure 2E). Therefore, NKG7 may serve as a valuable phenotypic marker to capture CD4‐CTLs.

### 2.3 NKG7 Correlates with Cytotoxicity‐Associated Transcripts Across Disparate Tissues

**FIGURE 3:**
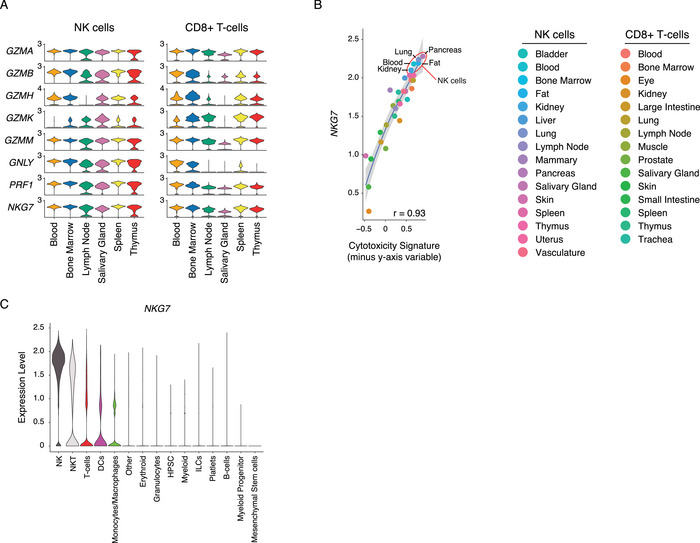
NKG7 correlates with cytotoxicity across organs. (A) Violin plots showing the gene expression of cytotoxic molecules in NK (left) and CD8+ T‐cells (right) across major immunological organs. (B) Scatter plots of the correlation between *NKG7* gene expression and cytotoxicity score within NK or CD8+ T‐cells across all organs of the Tabula Sapiens dataset. Pearson correlation is displayed and the shaded area represents the 95% CI. (C) Violin plots showing the expression of *NKG7* across immune populations within the Tabula Sapiens dataset.

It was previously observed that *NKG7* strongly correlates with cytotoxicity in healthy PBMC subsets. However, the tissue‐specific expression patterns of *NKG7* and other cytotoxic molecule genes were poorly described. To address this, we took the Tabula Sapiens scRNAseq dataset and probed the expression of cytotoxic genes across NK and CD8+ T‐cell populations from various immunologically relevant organs. This revealed that *NKG7* is consistently expressed across NK cells from various tissue locations (Figure 3A). In contrast, genes such as *GZMH* or *GZMK* showed dynamic expression differences between NK cells from different tissues. This possibly reflects known and expected differences in the abundance of NK subsets in these tissues and their distinct expression profiles of *GZMH* and *GZMK*.

Similarly, in CD8+ T‐cells, *NKG7* was consistently detected, although there was a notable drop in expression observed in salivary gland CD8+ T‐cells. Across both NK and CD8+ T‐cells, *GZMA*, *GZMM*, *PRF1*, and *NKG7* were consistently expressed and detected regardless of tissue. Importantly, *GZMB* had variable expression within CD8+ T‐cells and was minimally detected in lymph nodes, salivary glands, and thymic tissues. As such, this robust expression of *NKG7* extended across all tissues of the Tabula Sapiens dataset and *NKG7* gene expression was found to be strongly correlated (*r* = 0.93) with the cytotoxicity signature within NK and CD8+ T cells from various tissues (Figure 3B). In contrast, *GZMB* showed a poorer correlation with cytotoxicity‐associated transcripts across tissues with a Pearson *r* = 0.72 (data not shown). As mentioned, in this dataset *NKG7* was found primarily in NK, NKT cells, and T cells, while a low expression level was observed in DCs and monocytes (Figure 3C). Altogether, these data demonstrate that NKG7 captures potential cytotoxic activity within traditionally cytotoxic populations regardless of the tissue of origin.

### 2.4 NKG7 is Consistently Expressed in Cells Co‐Expressing Multiple Cytotoxicity‐Associated Transcripts

While the expression of cytotoxic molecules is commonly assessed in both flow cytometry and single‐cell sequencing, their co‐expression at single‐cell level is often overlooked in downstream analyses. Therefore, we aimed to characterize the co‐expression patterns of cytotoxicity‐associated transcripts, utilizing single‐cell data.

**FIGURE 4:**
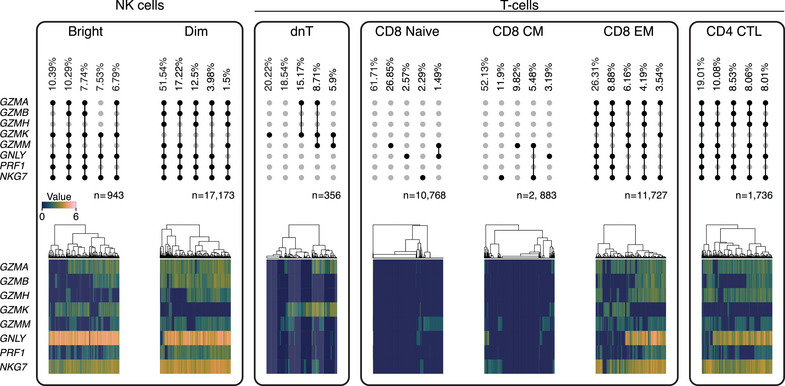
NKG7 is consistently co‐expressed with other cytotoxic molecules. Co‐expression pattern of cytotoxic molecules across NK and T‐cell subsets. UpSet plots (top row) demonstrate the co‐expression pattern and frequency observed. Meanwhile, heatmaps (bottom row) demonstrate the expression profile of individual cells. *n* value represents the number of cells of a particular subset included in the analysis.

We observed that a considerable proportion (∼27%) of proinflammatory NK‐Bright cells co‐expressed *GZMA*, *GZMK*, *GNLY*, *PRF1*, and *NKG7* (Figure 4). In contrast, the cytotoxic NK‐Dim subset consistently co‐expressed *GZMA*, *GZMB*, *GNLY*, *PRF1*, and *NKG7*, with subsets differing in their co‐expression of *GZMH* and *GZMM*. Interestingly, circa 20% of dnT cells were observed to exhibit solitary expression of *GZMK*, while a small subset of dnT cells co‐expressed *GZMK* with either *GZMA*, *GZMM*, or both. An appreciable frequency of CD8‐Naïve and CD8‐CM population subsets are characterized by the singular expression of cytotoxic molecules, even though these subsets are largely not cytotoxic. In contrast, cytotoxic subsets such as CD8‐EM and CD4‐CTLs demonstrated diverse co‐expression patterns. For example, while the top five most abundant co‐expression patterns for CD8‐EM and CD4‐CTLs consistently contained *GZMA* and *NKG7*, there was variable usage of other cytotoxic molecules. Within CD8‐EM cells, all remaining cytotoxic molecules were variably expressed. However, within CD4‐CTLs, there was consistent expression of *GZMH* and *GNLY*, while *GZMK* was absent from the top five most abundant CD4‐CTL cell patterns. Notably, three of the top five most abundant CD8‐EM and CD4‐CTL patterns did not contain *PRF1*. Additionally, among cytotoxic populations (NK‐Bright, NK‐Dim, CD8‐EM, and CD4‐CTL), *NKG7*‐negative phenotypes were only observed at low frequency within the CD8‐EM subset. This reiterates the abundant expression of *NKG7* in cytotoxicity‐associated populations. These findings further highlight that *NKG7* is consistently observed across immune cell subsets that co‐express multiple cytotoxicity‐associated transcripts.

### 2.5 NKG7 Identifies Cytotoxic Tumor‐Infiltrating Cells

**FIGURE 5:**
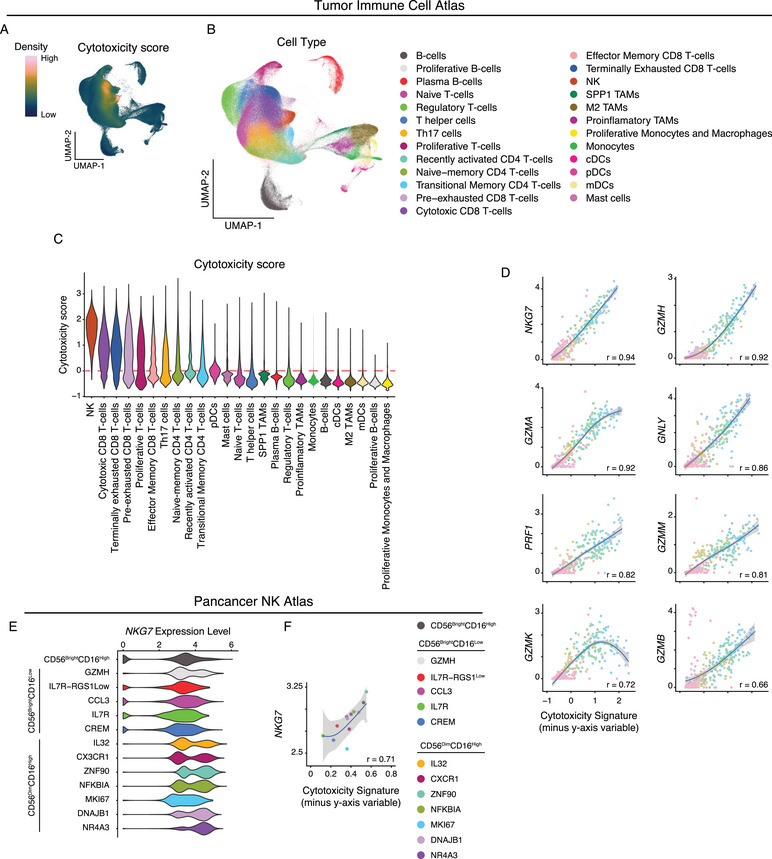
NKG7 correlates with cytotoxicity in tumor‐infiltrating immune subsets. (A) Density of cytotoxicity score expression overlaid on UMAP coordinates of the tumor immune cell atlas. (B) UMAP plot of the different cell subsets identified within the tumor immune cell atlas. (C) Violin plots showing the cytotoxicity score of various cell subsets within tumor immune cell atlas. (D) Scatterplot of the correlation between *y*‐axis gene expression and cytotoxicity signature. Pearson correlation is displayed and the shaded area represents the 95% CI. (E) UMAP plot highlighting the NK subsets identified within the pancancer NK atlas (left). The violin plot depicts the expression of *NKG7* across various NK subsets within the pancancer NK atlas. (F) Scatterplot of the correlation between *NKG7* or Granzyme B (*GZMB*) expression and cytotoxicity score across NK subsets. Pearson correlation is displayed and the shaded area represents the 95% CI.

After showing that *NKG7* is consistently expressed across tissues and a core constitute of the polyfunctional cytotoxic program, we next sought to evaluate cytotoxic molecule expression dynamics in tumor‐infiltrating immune cells. It is indeed known that the tumor microenvironment (TME) can drastically alter expression patterns of infiltrating cytotoxic immune cells. To investigate this, we utilized the Tumor Immune Cell Atlas [30]. This atlas contains tumor‐infiltrating cells (*n* = 314,679) from 177 patients spanning 12 tumor subtypes (Figure S2A). Looking at *NKG7* expression, we found high expression levels in NK cells, and CD8+ T cell subsets, including exhausted, cytotoxic, and EM populations (Figure S2B). We then scored cells within the dataset for overall cytotoxicity and identified two areas of dense signal, corresponding to the CD8 T‐cell and NK cell populations (Figure 5A–C). Interestingly, cells annotated as terminally exhausted CD8 T‐cells show high levels of expression of cytotoxicity genes. Consistent with previous observations, *NKG7* gene expression strongly correlates with the overall cytotoxicity signature (Figure 5D). Indeed, *NKG7* is the strongest correlate of cytotoxicity across tumor‐infiltrating immune cells. Surprisingly, *GZMH* showed the second highest correlation with cytotoxicity while *GZMB* scored poorly. The low correlation of *GZMB* expression with the overall cytotoxicity signature is driven by the unique and singular expression of *GZMB* in pDC cells. However, even in the absence of pDC cells, *GZMB* expression was a poorer correlate of cytotoxicity score than *NKG7* gene expression (Figure S2C). NK cells are a potent cytotoxic population but are poorly captured within the Tumor Immune Cell Atlas (*n* = 9496). Therefore, we utilized a pancancer NK cell atlas (*n* = 34,900) to further investigate cytotoxic molecule expression within tumor‐infiltrating NK cells [31]. The NK cell subsets referenced in this study were predefined within the pancancer NK cell atlas dataset. Within these tumor‐infiltrating NK subsets, NKG7 was consistently expressed (Figure 5E). Additionally, *NKG7* was highly expressed in tumor‐infiltrating NK cells from all disease subsets analyzed (Figure S2D). As such, *NKG7* gene expression positively correlated with the overall cytotoxicity signature in tumor‐infiltrating NK cells (Figure 5F). *NKG7* was a stronger correlate for cytotoxicity than all other markers except *GZMA* and *PRF1* (Figure S2E). Taken together, these data demonstrate that *NKG7* expression is maintained on tumor‐infiltrating immune cells in numerous malignancies. Furthermore, *NKG7* positively correlates with overall cytotoxicity, outperforming traditional markers of cytotoxicity such as *GZMB*.

## 3 Discussion

NKG7 is an emerging component of the cytotoxic machinery, yet its functional roles and expression dynamics remain poorly understood. To address this gap, we conducted a comprehensive analysis of NKG7 expression alongside other core components of cytotoxic granules, providing insights into its reliability as a cytotoxicity marker across diverse contexts. As such, in our study, NKG7 emerged as a robust and reliable marker of cytotoxicity across healthy and disease contexts. Our analyses revealed that NKG7 is consistently expressed across various cytotoxic immune subsets and immunologically relevant tissues. Notably, *NKG7* demonstrated superior reliability compared with traditional markers such as *GZMB* and *PRF1*. Indeed, our analyses showed that *NKG7* is consistently detected across immune cell subsets that are characterized by the expression of multiple cytotoxicity‐associated transcripts. For example, while plasmacytoid dendritic cells (pDCs) express solitary *GZMB*, this alone does not reflect engagement with the broader cytotoxic program. In contrast, *NKG7* expression correlated consistently with cytotoxicity‐associated signatures across immune subsets.

Cytotoxic tumor‐infiltrating lymphocytes (TILs) are critical determinants of patient prognosis, with higher frequencies consistently correlating with improved survival across malignancies [32]. Transcript‐based signatures, such as the cytolytic activity score (*GZMA*+ *PRF1*+), have been developed to quantify TIL cytotoxicity and predict disease outcomes [33, 34, 35]. Notably, we show that NKG7 outperforms many traditional markers, offering improved resolution in capturing functional cytotoxicity. In line with this, *NKG7* expression has been reported by other studies to be associated with favorable clinical outcomes and to be important for effective T‐cell immunity [14, 16, 18, 36]. This growing recognition underscores the importance of understanding NKG7's integration into broader cytotoxicity programs.

Our analysis revealed the underappreciated heterogeneity of cytotoxic molecule usage. For instance, proinflammatory (NK‐bright) and cytotoxic (NK‐Dim) cells exhibit distinct usage of *GZMK* and *GZMH*, suggesting a potential functional specialization within cytotoxic programs [37]. Similarly, tissue‐specific cytotoxic programs in NK and CD8+ T cells demonstrated substantial variability in granzyme expression, which likely reflects differences in subset composition and tissue reprogramming. For example, differences in granzyme expression among NK subsets may reflect tissue‐resident programming, while the maturation state influences granzyme patterns in CD8+ T cells. Despite these variations, NKG7 expression remained consistent across tissues and subsets, further supporting its role as a universal cytotoxicity marker.

Our results from single‐cell analysis revealed cytotoxic cells lacking *PRF1*, suggesting potential alternative pathways for granzyme activity. Mechanisms mediating granzyme entry, such as mannose‐6‐phosphate receptor or serglycin‐mediated transport, may underlie these observations [38, 39]. Notably, we showed that *NKG7* expression was preserved in *PRF1*‐negative populations, suggesting its potential role in cytotoxic programs that operate independently of PRF1. However, the extent to which these pathways operate independently of PRF remains unclear. Experimental evidence from others indicates that perforin (and not granzymes) is essential for effective tumor control [40]. This suggests that perforin‐negative populations may be leveraging granzymes for noncytotoxic roles, such as extracellular matrix (ECM) remodeling. Granzymes contribute to ECM remodeling by cleaving proteins such as fibronectin and laminin, with complex effects on tumor progression. While ECM degradation can enhance immune infiltration and induce anoikis, it may also promote metastasis by weakening cell adhesion. Additionally, ECM breakdown releases immunomodulatory cytokines and chemotactic fragments [41], demonstrating the dual roles of granzymes in immune regulation and cancer progression. Despite this complexity, our data show that *NKG7* is consistently co‐expressed with cytotoxicity‐associated transcripts, including in *PRF1*‐negative populations. This suggests that *NKG7* may also mark immune cells involved in *PRF1*‐independent mechanisms. However, whether these cells are functionally cytotoxic or performing noncytotoxic processes, remains to be determined.

*NKG7* consistently emerged as a central component of the cytotoxic molecule expression program across NK subsets and effector states such as CD8‐EM and CD4‐CTL, identifying cytotoxic cells regardless of their molecular programs. *NKG7* also functioned to demarcate CD4‐CTLs, a subset with emerging relevance in antitumor responses. Indeed, *NKG7* is frequently observed in gene signatures of CD4‐CTLs across numerous disease contexts [29, 42–46]. Additionally, *NKG7* was consistently highly correlated with cytotoxicity within TILs, surpassing other canonical markers of cytotoxicity. This underscores its stability and reliability as an effective correlate of cytotoxicity signature in T cells, even in the immunosuppressive tumor microenvironment.

Interestingly, while our results showed that *NKG7* strongly correlates with cytotoxicity‐associated transcripts in NK cells under steady‐state conditions, the usage of these transcripts appears to differ in tumor‐infiltrating NK cells. Indeed, within tumor‐infiltrating NK cells, *NKG7*—while highly expressed and a strong correlate of cytotoxicity signature—was surpassed by *GZMA* and *PRF1* in terms of correlation with cytotoxicity score. This observation highlights the dynamic regulation of cytotoxicity‐associated gene usage across tissues and pathological states.

While our findings indicate that *NKG7* expression is strongly correlated with a broader cytotoxicity gene program, direct functional experimentation evaluating the cytotoxic capacity of NKG7‐expressing cells is required. Indeed, functional insights into NKG7's role in cytotoxicity are in their infancy and require further investigation. In addition, single‐cell RNAseq is subject to technical limitations such as transcript dropout and gene‐specific detection biases. As a result, it is not possible to fully distinguish whether the consistent detection of *NKG7* reflects true biological abundance or enhanced technical detectability. As such, interpretations of *NKG7* expression patterns should therefore consider both biological and technical contributions.

Regardless, our results demonstrate that *NKG7* is a central and reliable marker of cytotoxicity‐associated transcriptional programs. The consistent expression across diverse immune subsets, tissues, and disease contexts underscores *NKG7*s utility as a robust cytotoxicity correlate. Furthermore, the detection of *NKG7* in *PRF1*‐negative populations raises intriguing possibilities about *NKG7*’s involvement in alternative cytotoxic or immunomodulatory pathways, warranting deeper mechanistic investigation. While the precise functional role of *NKG7* remains to be fully elucidated, the consistent association with cytotoxic gene signatures across both steady‐state and pathological conditions supports its value as a robust tool for identifying cells with cytotoxic potential. These findings position *NKG7* as a useful marker for advancing our understanding of immune effector function as a potential translational biomarker in studies of immune‐mediated disease and cancer.

## 4 Methods

### 4.1 Cell Culture and Transfection of HEK293T Cells

Human embryonic kidney 293 cells (HEK293T) were cultured in DMEM medium, containing GLUTAMax (Gibco, cat. 61965‐026), supplemented with 10% (vol/vol) Fetal Bovine Serum (FBS) (Gibco, cat. A5256801) and 100 units/mL of penicillin G and 100 ug/mL of streptomycin sulfate (Gibco, cat. 15140‐122) at 37°C, 5% CO_2_.

For the transfection, HEK293T cells were seeded in a six‐well plate (1 × 10^6^ cells/well) and incubated overnight. On the next day, cells were transfected via the calcium phosphate method, as previously described [47], so that they could overexpress mNeon‐NKG7 or 3xFLAG‐TIA‐1. Plasmids were purchased from BioCat and sequences can be found as following: mNeonGreen‐Linker‐NKG7(165aa), cloning vector pcDNA3.0, cloning sites NotI(GCGGCCGC)‐ XhoI(CTCGAG); sequence: GCGGCCGCCACCATGGTGAGCAAGGGCGAGGAGGATAACGCCTCTCTCCCAGCGACACATGAGTTACACATCTTTGGCTCCATCAACGGTGTGGACTTTGACATGGTGGGTCAGGGCACCGGCAATCCAAATGATGGTTATGAGGAGTTAAACCTGAAGTCCACCAAGGGTGACCTCCAGTTCTCCCCCTGGATTCTGGTCCCTCATATCGGGTATGGCTTCCATCAGTACCTGCCCTACCCTGACGGGATGTCGCCTTTCCAGGCCGCCATGGTAGATGGCTCCGGATACCAAGTCCATCGCACAATGCAGTTTGAAGATGGTGCCTCCCTTACTGTTAACTACCGCTACACCTACGAGGGAAGCCACATCAAAGGAGAGGCCCAGGTGAAGGGGACTGGTTTCCCTGCTGACGGTCCTGTGATGACCAACTCGCTGACCGCTGCGGACTGGTGCAGGTCGAAGAAGACTTACCCCAACGACAAAACCATCATCAGTACCTTTAAGTGGAGTTACACCACTGGAAATGGCAAGCGCTACCGGAGCACTGCGCGGACCACCTACACCTTTGCCAAGCCAATGGCGGCTAACTATCTGAAGAACCAGCCGATGTACGTGTTCCGTAAGACGGAGCTCAAGCACTCCAAGACCGAGCTCAACTTCAAGGAGTGGCAAAAGGCCTTTACCGATGTGATGGGCATGGACGAGCTGTACAAGGGGTCTGGTGGCAGTGGAGGGGGATCCATGGAGCTCTGCCGGTCCCTGGCCCTGCTGGGGGGCTCCCTGGGCCTGATGTTCTGCCTGATTGCTTTGAGCACCGATTTCTGGTTTGAGGCTGTGGGTCCCACCCACTCAGCTCACTCGGGCCTCTGGCCAACAGGGCATGGGGACATCATATCAGGCTACATCCACGTGACGCAGACCTTCAGCATTATGGCTGTTCTGTGGGCCCTGGTGTCCGTGAGCTTCCTGGTCCTGTCCTGCTTCCCCTCACTGTTCCCCCCAGGCCACGGCCCGCTTGTCTCAACCACCGCAGCCTTTGCTGCAGCCATCTCCATGGTGGTGGCCATGGCGGTGTACACCAGCGAGCGGTGGGACCAGCCTCCACACCCCCAGATCCAGACCTTCTTCTCCTGGTCCTTCTACCTGGGCTGGGTCTCAGCTATCCTCTTGCTCTGTACAGGTGCCCTGAGCCTGGGTGCTCACTGTGGCGGTCCCCGTCCTGGCTATGAAACCTTGTGACTCGAG3xFLAG‐Linker‐TIA1(386aa), cloning vectors: pcDNA3.0, cloning sites NotI(GCGGCCGC)‐ XhoI(CTCGAG); sequence: GCGGCCGCCACCATGGACTATAAGGACCACGACGGAGACTACAAGGATCATGATATTGATTACAAAGACGATGACGATAAGGGGTCTGGTGGCAGTGGAGGGGGATCCATGGAGGACGAGATGCCCAAGACTCTATACGTCGGTAACCTTTCCAGAGATGTGACAGAAGCTCTAATTCTGCAACTCTTTAGCCAGATTGGACCTTGTAAAAACTGCAAAATGATTATGGATACAGCTGGAAATGATCCCTATTGTTTTGTGGAGTTTCATGAGCATCGTCATGCAGCTGCAGCATTAGCTGCTATGAATGGACGGAAGATAATGGGTAAGGAAGTCAAAGTGAATTGGGCAACAACCCCTAGCAGTCAAAAGAAAGATACAAGCAGTAGTACCGTTGTCAGCACACAGCGTTCACAAGATCATTTCCATGTCTTTGTTGGTGATCTCAGCCCAGAAATTACAACTGAAGATATAAAAGCTGCTTTTGCACCATTTGGAAGAATATCAGATGCCCGAGTGGTAAAAGACATGGCAACAGGAAAGTCTAAGGGATATGGCTTTGTCTCCTTTTTCAACAAATGGGATGCTGAAAACGCCATTCAACAGATGGGTGGCCAGTGGCTTGGTGGAAGACAAATCAGAACTAACTGGGCAACCCGAAAGCCTCCCGCTCCAAAGAGTACATATGAGTCAAATACCAAACAGCTATCATATGATGAGGTTGTAAATCAGTCTAGTCCAAGCAACTGTACTGTATACTGTGGAGGTGTTACTTCTGGGCTAACAGAACAACTAATGCGTCAGACTTTTTCACCATTTGGACAAATAATGGAAATTCGAGTCTTTCCAGATAAAGGATATTCATTTGTTCGGTTCAATTCCCATGAAAGTGCAGCACATGCAATTGTTTCTGTTAATGGTACTACCATTGAAGGTCATGTTGTGAAATGCTATTGGGGCAAAGAAACTCTTGATATGATAAATCCCGTGCAACAGCAGAATCAAATTGGATATCCCCAACCTTATGGCCAGTGGGGCCAGTGGTATGGAAATGCACAACAAATTGGCCAGTATATGCCTAATGGTTGGCAAGTTCCTGCATATGGAATGTATGGCCAGGCATGGAACCAGCAAGGATTTAATCAGACACAGTCTTCTGCACCATGGATGGGACCAAATTATGGAGTGCAACCGCCTCAAGGGCAAAATGGCAGCATGTTGCCCAATCAGCCTTCTGGGTATCGAGTGGCAGGGTATGAAACCCAGTGACTCGAG. Cells were collected after 24 h from the transfection, washed twice in PBS and then lysed to extract proteins, as described in the next paragraph.

### 4.2 Western Blots

Cells were lysed using RIPA lysis buffer supplemented with protease and phosphatase inhibitors diluted 1:100 (cat. 5872, Cell Signaling Technology). The total protein concentration was determined with the Protein Assay Dye Reagent (cat. 5000006, Bio‐Rad,). For each sample, 20 µg of protein were prepared in Laemmli buffer and separated on 15 % polyacrylamide gels via SDS‐PAGE. After electrophoresis, proteins were transferred to nitrocellulose blotting membranes (cat. 10600004, Cytiva) in a wet blotting Mini Trans‐Blot Cell system (Bio‐Rad). The membranes were incubated with 5 % (w/v) bovine serum albumin (BSA) (cat. 8076, Carl Roth) in TRIS‐buffered saline with 0.05 % (v/v) of Tween 20 (TBS‐T) for 1 h at room temperature followed by incubation with primary antibodies overnight at 4°C. All primary antibodies were diluted in 5 % (w/v) BSA in TBS‐T with following dilutions: anti‐NKG7 (clone 2G9A10F5, 1:200, cat. IM2550, Beckman Coulter), anti‐TIA‐1 (clone EPR9304, 1:1000, cat. ab140595, abcam), anti‐FLAG (clone M2, 1:500, cat. F3165, Sigma‐Aldrich), anti‐β‐actin (clone 13E5, 1:1000, cat. 4970, Cell Signaling Technology), anti‐mNeonGreen (clone 32F6, 1:200, cat. 32f6, Proteintech), anti‐NKG7 (polyclonal, 1:1000, cat. 65507, Cell Signaling Technology), anti‐FLAG (clone L5, 1:500, cat. NBP1‐06712, Novus Biologicals), anti‐mNeonGreen (polyclonal, 1:1000, cat. 53061, Cell Signaling Technology). After overnight incubation with primary antibodies, the membranes were washed three times with TBS‐T for 5 min each and incubated with secondary antibodies for 1 h at room temperature. All secondary antibodies were used 1:15000 diluted in 5 % (w/v) BSA in TBS‐T: IRDye 800CW Goat anti‐Mouse (cat. 926–32210, LI‐COR), IRDye 800CW Donkey anti‐Rabbit (cat. 926–32213, LI‐COR), IRDye 680RD Goat anti‐Rat (cat. 926–68076, LI‐COR), IRDye 680RD Goat anti‐Rabbit (cat. 926–68071, LI‐COR), and IRDye 680RD Donkey anti‐Mouse (cat. 926–68072, LI‐COR). After incubation with secondary antibodies, the membranes were washed three times with TBS‐T for 5 min each and the protein bands were imaged using the Odyssey CLx system (LI‐COR).

### 4.3 Peripheral Blood Mononuclear Cell Dataset

#### 4.3.1 Dataset and Preprocessing

The PBMC dataset published and described in [25] was downloaded from https://atlas.fredhutch.org/nygc/multimodal‐pbmc/. This dataset contains PBMCs from eight healthy donors collected at three time points relative to HIV vaccination: prevaccination, 3 days postvaccination, and 7 days postvaccination. In total, it includes 161,764 cells and 20,957 features, comprising RNA expression and 228 CITE‐seq antibody‐derived tags (ADTs).

The original study performed quality control, cell type annotation, and multimodal integration, using both RNA and ADT data for cell type identification and dimensionality reduction (see [25] for details). For our analysis, we focused only on cells from the prevaccination time point. Cells annotated as doublets at the celltype.l1 annotation level were removed, reducing the dataset to 161,159 cells. All downstream analyses utilized the dimensionality reduction and cell type classifications provided by the original dataset authors.

#### 4.3.2 Custom Cell Type Annotation

The dataset authors provided cell type annotations at multiple levels of resolution, with celltype.l1 representing broad cell classifications and celltype.l3 offering more detailed subtype annotations [25]. To simplify and standardize labeling, we re‐annotated the dataset by consolidating celltype.l3 categories into broader, more interpretable groups. This postprocessing step retained the structure of the original dataset but grouped similar populations under unified labels. Specifically, the following groupings were applied. All B‐cell subtypes, including “B intermediate lambda,” “B naive kappa,” “B intermediate kappa,” “B memory kappa,” “B naive lambda,” and “B memory lambda,” were combined into a single category labeled “B_cells.” The “Plasma” and “Plasmablast” subtypes were grouped together as “Plasmablasts.” The NK subsets labeled “NK_1” through “NK_4” were merged into a single category called “NK_Dim,” while “Treg Naive” and “Treg Memory” were combined under the label “Treg.” The “dnT_1” and “dnT_2” subsets were consolidated into the “dnT” category. Similarly, the dendritic cell subsets “cDC2_1,” “cDC2_2,” and “ASDC_mDC” were grouped as “cDC2,” while “ASDC_pDC” and “pDC” were merged into the “pDC” category. The gamma‐delta T‐cell subsets “gdT_2,” “gdT_3,” and “gdT_4” were combined into a single “gdT” category, whereas “gdT_1” was retained as “gdT_V9D2.”

For CD4+ T‐cell subsets, “CD4 TCM_1” through “CD4 TCM_3” were grouped as “CD4_CM,” and “CD4 TEM_1” through “CD4 TEM_4” were combined as “CD4_EM.” For CD8+ T cells, “CD8 Naive” and “CD8 Naive_2” were merged into “CD8_Naive,” while “CD8 TEM_1” through “CD8 TEM_6” were consolidated under “CD8_EM.” Additionally, “CD8 TCM_1” through “CD8 TCM_3” were grouped as “CD8_CM.” To maintain consistency, formatting adjustments were applied, including replacing spaces with underscores and standardizing nomenclature for “Platelets” and “Prolif” (proliferating cells). This postprocessing step resulted in 28 distinct cell populations used for downstream analyses.

### 4.4 Tabula Sapiens Dataset

#### 4.4.1 Dataset and Preprocessing

The TS_immune dataset from the Tabula Sapiens project was downloaded from figshare (https://figshare.com/projects/Tabula_Sapiens/100973). This dataset is a comprehensive single‐cell RNA sequencing atlas of 58,870 genes across 264,824 cells from 24 different anatomical sites [48]. These data were derived from both 10x Genomics and Smart‐seq2 technologies, with the majority of cells originating from 10x Genomics. For this analysis, we filtered the data to retain only cells processed with 10x Genomics, resulting in 249,961 cells. Donors with a low cell count (fewer than 50 cells) were excluded, specifically TSP3 (2 cells), TSP12 (48 cells), and TSP13 (0 cells), leaving 12 donors in the final dataset.

#### 4.4.2 Data Annotation and Cleaning

The cell type annotations provided with the dataset were cleaned and formatted to resolve inconsistencies and correct misspellings. Details of this cleaning process are available in the GitHub repository associated with this manuscript. See code availability for more information.

#### 4.4.3 Normalization and Integration

The dataset was split into individual layers by donor, and data normalization was then performed on each donor separately. Data normalization was performed using the SCTransform function (vst.flavor = “v2”, method = glmGamPoi). Principal component analysis (PCA) was conducted using RunPCA with default parameters. The datasets were then integrated using the IntegrateLayers function with canonical correlation analysis (CCA) as the integration method. This step utilized PCA dimensions from the SCTransform‐normalized data.

#### 4.4.4 Dimensionality Reduction and Imputation

Uniform manifold approximation and projection (UMAP) was calculated using RunUMAP on 30 dimensions of the integrated CCA reduction. For specific visualizations, imputed values were used to enhance interpretability. Imputation was performed using RunALRA, which increased the proportion of nonzero entries in the data matrix from 16.19% to 49.97%.

### 4.5 Tumor Immune Cell Atlas

The tumor immune cell atlas dataset was downloaded from Zenodo (https://zenodo.org/records/4263972) and is described in detail in the accompanying publication [30]. The dataset originally contained 92,256 features across 317,111 cells, representing 13 tumor types from 181 patients and annotated with 25 cell types. For the analysis, cells from the ovarian cancer (OC) subtype were removed due to the low number of cells available (2432 total). Consequently, the analyzed dataset included data from 177 patients across 12 tumor subtypes.

### 4.6 Pancancer NK atlas

The NK Atlas dataset was downloaded from Zenodo (https://zenodo.org/records/8275845) and contained expression data for 13,493 genes across 142,304 cells and is described in detail in the accompanying publication [31]. Data were filtered to include only tumor‐derived cells, resulting in a final dataset of 34,900 cells across 24 tumor types and 13 NK subtypes as defined in the original dataset.

### 4.7 Software and Versions

All analyses were conducted using the R programming environment (v4.4.0) [49] on a platform of x86_64‐apple‐darwin20 running macOS Ventura 13.0. The primary tools included the Seurat package (v5.1.0) [50] for data normalization, integration, dimensionality reduction, and visualization, with additional functionality provided by SeuratDisk (v0.0.0.902) [51], SeuratWrappers (v0.3.2) [52], and SeuratObject (v5.0.2) [53].

Visualization and data processing were performed using ggplot2 (v3.4.4) [54], dplyr (v1.1.4) [55], scCustomize (v2.1.2) [56], and Nebulosa (v1.14.0) [57]. Heatmaps and density plots utilized the “batlow” color scheme, accessed through the scico package (v1.5.0) [58].

### 4.8 Cytotoxicity Score and Correlation Analysis

Cytotoxicity score was calculated using the AddModuleScore function from the Seurat package, based on a predefined gene set consisting of *GZMA*, *GZMB*, *GZMH*, *GZMK*, *GZMM*, *GNLY*, *PRF1*, and *NKG7*.

For correlation analysis, expression data were first aggregated using the AggregateExpression function. Module score was calculated using AddModuleScore function across a series of gene sets generated by iteratively removing one marker at a time from the full cytotoxicity signature (see signature above). Pairwise correlation plots were generated using the FeatureScatter function to compare each module score with the expression level of the excluded marker.

### 4.9 Bulk RNAseq Dataset

The “Monaco” dataset [59] of RNA transcript abundance across human peripheral blood immune cells was downloaded from the Human Protein Atlas (https://www.proteinatlas.org). Data were plotted using GraphPad Prism (v10.0.3, GraphPad Software, Boston, Massachusetts USA, www.graphpad.com).

### 4.10 PBMCs Isolation

Peripheral blood from healthy donors was provided by the Institute for Experimental Hematology and Transfusion Medicine at the University Hospital Bonn, Bonn, Germany. PBMCs were isolated from peripheral blood by Ficoll‐Paque PLUS (Cytiva, cat. 17144003) density gradient, following the standard protocol. NK cells were isolated from PBMCs using the EasySep Human NK cell Isolation Kit (STEMCELL Technologies, cat. 17955). After being isolated, PBMCs and NK cells were processed for flow cytometry staining.

### 4.11 Flow Cytometry

Flow cytometry staining was performed in 96‐well round‐bottom microplates (cat 92697, TPP), at room temperature and protected from light. Cells were firstly washed twice in PBS, then incubated with 50 uL TruStain FcX (1:200, cat. 422302, BioLegend) and Live/Dead Blue Fixable dye (1:1000, cat. L23105, Invitrogen), for 15 min in room temperature. After washing twice in PBS cells were then incubated with 50 uL of a cocktail of fluorescence‐conjugated antibodies recognizing surface molecules, for 20 min, at room temperature. The cocktail for the surface staining contained the following antibodies at the indicated dilution: BUV395‐CD8 (clone RPA‐T8, 1:200, cat. 563796, BD Biosciences), BUV496‐CD16 (clone 3G8, 1:200, cat. 612944, BD Biosciences), BUV563‐CD56 (clone NCAM16, 1:200, cat. 612928, BD Biosciences), BV570‐CD45RA, (clone HI100, 1:100, cat. 304132, BioLegend), BV650‐CD4 (clone OKT4, 1:200, cat. 317435, BioLegend), BV785‐CD62L (clone DREG‐56, 1:200, cat. 304829, BioLegend), and APC‐Cy7‐CD3 (clone SK7, 1:100, cat. 557832, BD Biosciences). After the incubation with the antibody cocktail, samples were washed twice in PBS and then incubated with 100 uL of eBioscience Foxp3/Transcription Factor Staining Buffer Set (ThermoFisher, cat. 00‐5523‐00), for 15 min. Cells were then washed twice with wash buffer and incubated for 30 min with 50 uL of a cocktail of fluorescence‐conjugated antibodies reactive against intracellular targets. The cocktail contained the following antibodies: Pacific Blue‐Granzyme A (clone CB9, 1:50, cat. 507207, BioLegend), BV711‐Perforin (clone dG9, 1:50, cat. 308130, BioLegend), AF488‐Granzyme H (clone E3H7W, cat. 23455S, Cell Signaling Technologies), PE‐NKG7 (clone 2G9A10F5, 1:100, cat. IM3293, Beckman Coulter), PE‐Dazzle‐594‐Granzyme B (clone QA18A28, 1:50 cat. 396427, BioLegend), PE‐Cy7‐Granzyme K (clone GM26E7, 1:50, cat. 370515, BioLegend), AF647‐Granzyme M (clone 4B2G4, 1:50, cat. 566996, BD Biosciences), and AF700‐Granulysin (clone B‐L38, 1:50, cat. NBP3‐18104AF700, Novus Bio). As controls, a second antibody cocktail containing the following IgG controls: BV711‐Mouse IgG2k (clone MPC‐11, cat. 400354, BioLegend), AF88‐Rabbit IgG (polyclonal, cat. 4340S, Cell Signaling Technologies), PE‐Dazzle‐594‐Rat IgG1k (clone RTK2071, cat. 400445, BioLegend), PE‐Cy.7‐Mouse IgG1k (clone MOPC‐21, cat. 400125, BioLegend), AF647‐Mouse IgG1k (clone P3.6.2.8.1, cat. 51‐4714‐81, eBioscience), and AF700‐Mouse IgG1k (clone MOPC‐21, cat. 400143, BioLegend). After the incubation, all the samples were washed in FACS Buffer (PBS, 0.02% (vol/vol) FCS, 5 mM EDTA), and then fixed in 4% PFA (HistoFix, cat. P087.5, Roth). Cells were washed twice in FACS buffer and stored at +4°C, until acquisition. Samples were acquired on a Sony ID7000 7 lasers. Data were analyzed using FlowJo Software (v10.9.0, BD Life Sciences).

### 4.12 Statistical Analysis

Statistical analyses were performed using GraphPad Prism (v10.0.3, GraphPad Software) or R programming environment (v4.4.0) [49]. The specific statistical test used for each analysis are indicated in the corresponding figure legends.

### 4.13 Figure Preparation

Figures were arranged and formatted using Adobe Illustrator (v27.5, Adobe Inc.) and/or GraphPad Prism (v10.0.3, GraphPad Software)

## Author Contributions

**Roberta Turiello**: Conceptualization, validation, formal analysis, investigation, data curation, visualization, writing–original draft, and writing–review & editing. **Susanna S. Ng**: Conceptualization, validation, formal analysis, investigation, data curation, visualization, writing–original draft, and writing–review & editing. **Elisabeth Tan**: Investigation, data curation, and writing–review & editing. **Gemma van der Voort**: Investigation, data curation, and writing–review & editing. **Nazhifah Salim**: Investigation, data curation, and writing–review & editing. Michelle **C.R. Yong**: Investigation and writing–review & editing. **Malika Khassenova**: Methodology and investigation. Johannes Oldenburg: Resources. **Heiko Rühl**: Resources. **Jan Hasenauer**: Writing–review & editing. **Laura Surace**: Writing–review & editing. **Marieta Toma**: Resources. **Tobias Bald**: Writing–review & editing. **Michael Hölzel**: Conceptualization, supervision, project administration, funding acquisition, and writing–review & editing. **Dillon Corvino**: Conceptualization, software, formal analysis, data curation, visualization, supervision, project administration, writing–original draft, and writing–review & editing.

## Conflicts of Interest

The authors declare no conflicts of interest.

## Peer Review

The peer review history for this article is available at https://publons.com/publon/10.1002/eji.202551885.

## Supporting information




**Supporting file 1**: eji6002‐sup‐0001‐FigureS1.pdf

**Supporting file 2**: eji6002‐sup‐0002‐FigureS2.pdf

## Data Availability

The datasets analyzed in this study were obtained from https://www.proteinatlas.org (Bulk RNAseq dataset), https://atlas.fredhutch.org/nygc/multimodal‐pbmc/ (PBMC dataset), https://figshare.com/projects/Tabula_Sapiens/100973 (Tabula Sapiens dataset), https://zenodo.org/records/4263972 (Tumor Immune Cell Atlas dataset), and https://zenodo.org/records/8275845 (Pancancer NK Atlas dataset). All code used to generate figures can be found under the relevant repository at https://github.com/BaldLab and on Zenodo at 10.5281/zenodo.14865740. All other data are available upon request.
