# Supplementary figures and images for: NKG7 is a Stable Marker of Cytotoxicity Across Immune Contexts and Within the Tumor Microenvironment
Source: Eur J Immunol. 2025 Jun 20;55(6):e51885. doi: 10.1002/eji.202551885 (PMC12179582; doi:10.1002/eji.202551885)

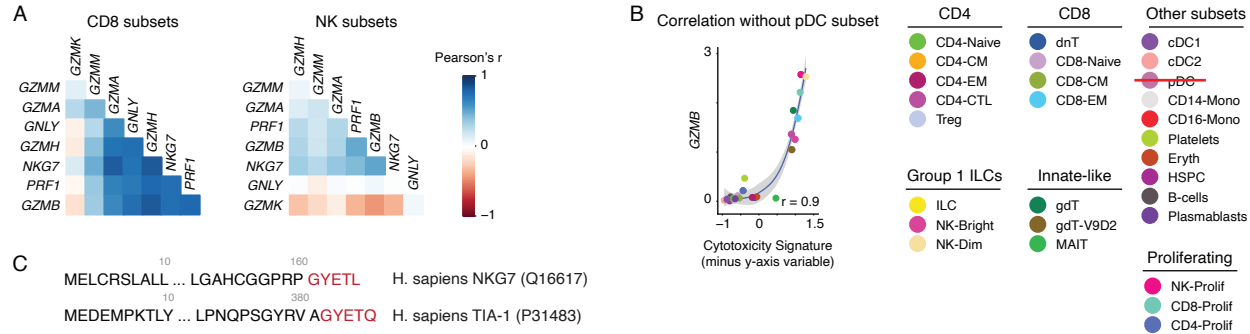

**C**

MELCRSLALL ... LGAHCGGPRP **GYETL** H. sapiens NKG7 (Q16617)

MEDEMPKTLTY ... LPNQPSGYRV **AGYETQ** H. sapiens TIA-1 (P31483)

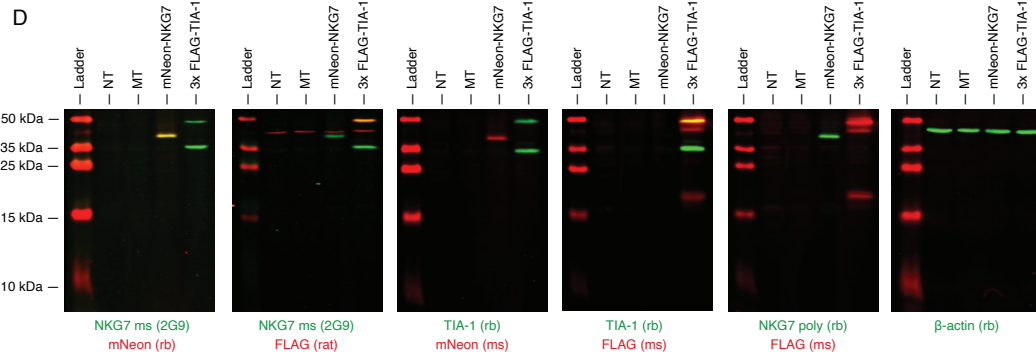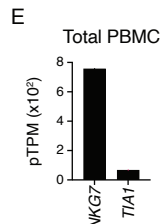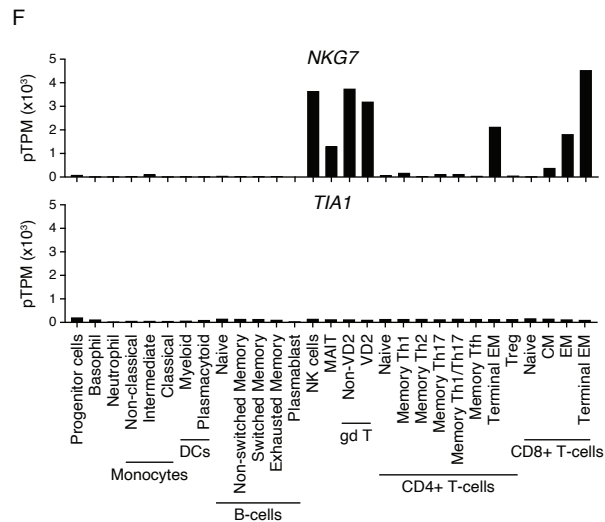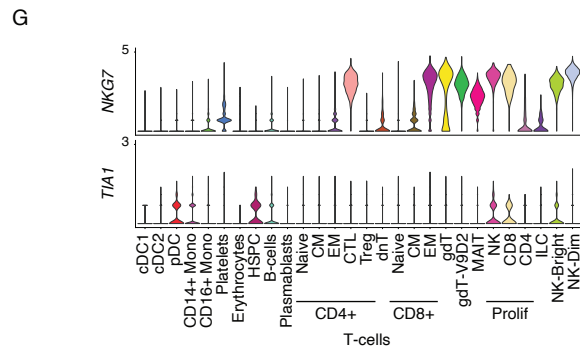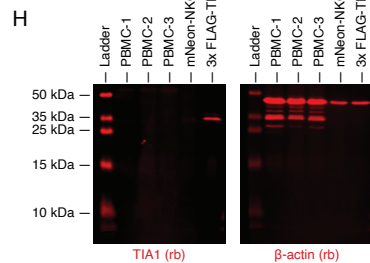

Supplement: Supplementary file 1 — Supporting file 1: eji6002‐sup‐0001‐FigureS1.pdf [file EJI-55-e51885-s002.pdf]

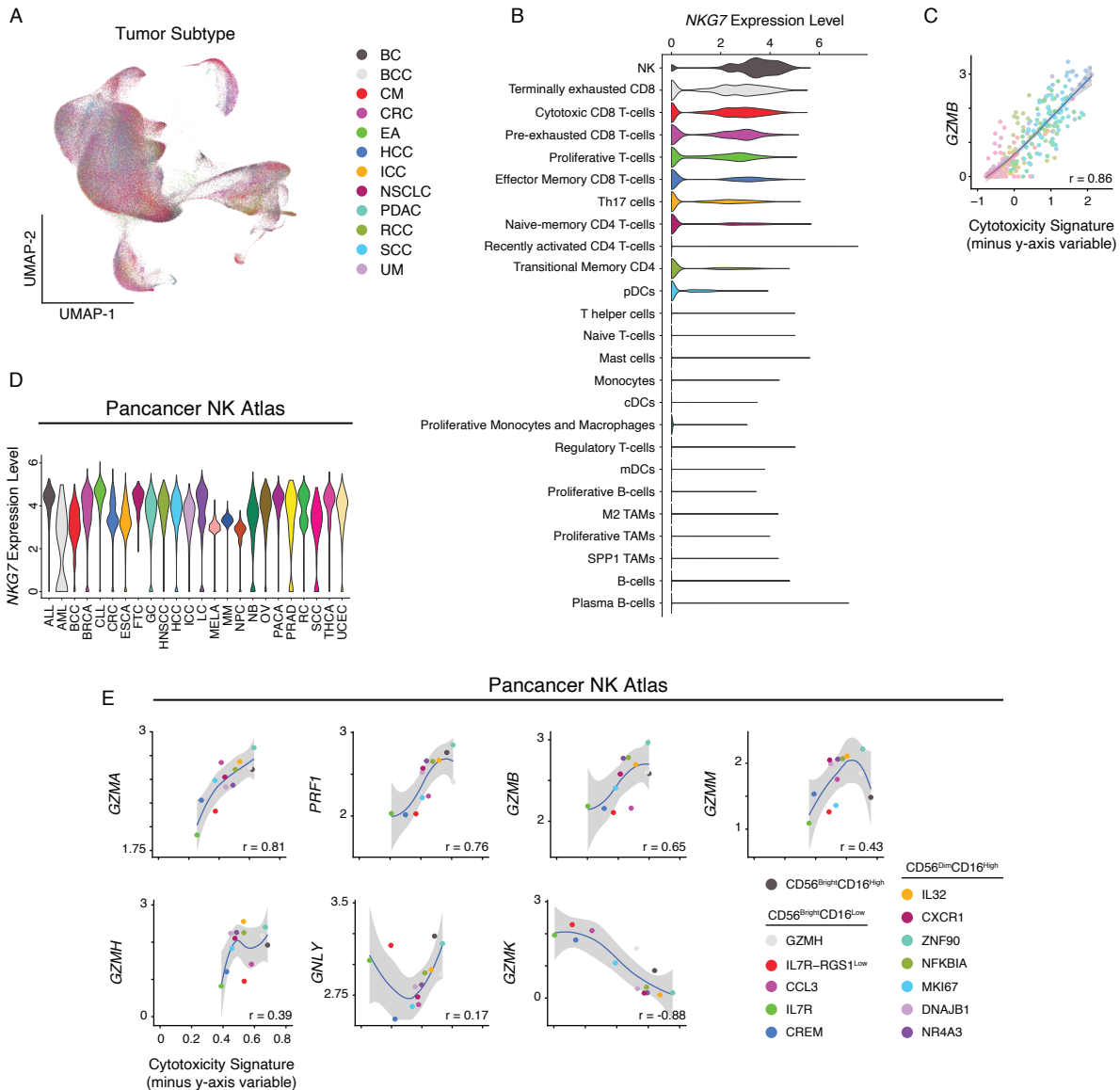

Supplement: Supplementary file 2 — Supporting file 2: eji6002‐sup‐0002‐FigureS2.pdf [file EJI-55-e51885-s001.pdf]
